# Esophageal Dysmotility Disorder and Dysphagia as Initial Manifestations of Lupus

**DOI:** 10.7759/cureus.46874

**Published:** 2023-10-11

**Authors:** Asmaa Hegazy, Fahad Aleidan, Saitah AlShammari, Raghad Alhindi, Shahid Bashir

**Affiliations:** 1 Internal Medicine, Rheumatology, King Saud Medical City (KSMC), Riyadh, SAU; 2 Medicine, Prince Sultan Military Medical City, Riyadh, SAU; 3 Internal Medicine, National Guard Hospital, Riyadh, SAU; 4 Neuroscience, Neuroscience Center, King Fahad Specialist Hospital - Dammam, Dammam, SAU

**Keywords:** autoimmune disease, manometry, motility disorders, dysphagia, systemic lupus erythematosus

## Abstract

Systemic lupus erythematosus (SLE) is a chronic, multisystemic autoimmune disease that is diagnosed using specific clinical and biochemical inclusion criteria. Here, we report the case of a young adult who was initially diagnosed with dysphagia and later diagnosed with SLE. Other rheumatological and non-rheumatological conditions were ruled out, and serology revealed that SLE was the cause of the patient’s symptoms.

To the best of our knowledge, no previous case studies or prior research identify dysphagia as a presenting symptom of SLE. While esophageal dysmotility ranging from ineffective esophageal motility (IEM) to achalasia can manifest as a later clinical consequence of SLE, it is rarely the first symptom of the disease. After diagnosis, the patient was treated with hydroxychloroquine and prednisolone, which led to significant improvement.

## Introduction

Systemic lupus erythematosus (SLE) is an inflammatory autoimmune disease that affects various body organs and may present with a wide range of early clinical symptoms and signs [1]. According to the European League Against Rheumatism/American College of Rheumatology (EULAR/ACR), gastrointestinal symptoms are not one of the clinical inclusion criteria of SLE. However, mouth ulcers are listed with a score of 2 under mucocutaneous clinical criteria [1].

When analyzing the published literature, it was found that a few case reports mentioned uncommon gastrointestinal tract involvement as the first sign of SLE in a variety of conditions, including peritonitis, acute acalculous cholecystitis, abdominal cocoon, and protein-losing enteropathy.

In our case, a 35-year-old woman with no history of comorbid illness or medication use presented to the emergency department (ED). Her only symptom was difficulty in swallowing.

## Case presentation

A single, nonsmoking, 35-year-old Saudi female presented to the ED. Her chief complaint was difficulty in swallowing. Her symptoms began three months before. She first experienced difficulty swallowing liquids, which later extended to solids as well. The condition was progressive, and her symptoms were alleviated by fasting. Furthermore, the presentation was characterized by subjective fever, a weight loss of 9 kg, pain while swallowing, recurrent oral ulcers, hair loss, and joint pain, primarily affecting the shoulders and knees.

The patient reported symptoms occurring one week before the presentation. The results of an outpatient esophagogastroduodenoscopy (EGD) were also unremarkable.

Otherwise, the patient denied similar episodes before and did not report a history of any genital ulcers, morning stiffness, skin changes, or other joint involvement. A systematic review of medical, surgical, family history, medication/herbal, or cultural methods history was unremarkable.

The patient appeared underweight; her body mass index (BMI) was 14. She was hemodynamically stable and had no lymphadenopathy or swelling in the neck. No skin changes or specific patterns of hair loss were observed, and a systematic examination was otherwise unremarkable.

The initial laboratory findings, including inflammatory markers, were mostly normal (Appendix). She presented with bicytopenia, displaying low hemoglobin and low WBC count; a Coombs test was positive (Table 1).

**Table 1:** Laboratory results of a 35-year-old female presented with difficulty swallowing. Ig, immunoglobulin; ANA, antinuclear antibody; Anti-DNA AB, anti-DNA antibody; MCV, mean corpuscular volume; MCH, mean corpuscular hemoglobin

Test	Result	Normal level
White blood cells (L^-1^)	3.4 × 10^9^	4-10
Platelet (L^-1^)	266 × 10^9^	150-400
Hemoglobin (g/dL)	8.9	12-15
Creatinine (mmol/L)	27	44-80
24-hour urine protein	0.2064	
ANA	1/1,280	Negative
Anti-DNA AB	Negative	Negative
C3 (g/L)	0.33	0.83-1.93
C4 (g/L)	0.078	0.15-0.57
B2-Glycoprotein 1 IgG	Negative	Negative
B2-Glycoprotein 1 IgM	Negative	Negative
Anti-cardiolipin IgG	Negative	Negative
Protease 3 antibodies	Negative	Negative
Fibrinogen (g/dL)	3.7	2-4
MCV (fL)	82.7	79-97
MCH (pg)	25.7	27-32
Anti-Smith	Negative	Negative <0.9
Anti-RO (SSA)	Positive	Negative <0.9
Anti-SSB-La	Negative	Negative <0.9
RF	<7 IU/mL	Negative
C-reactive protein	3.53 mg/L	0-4
Direct Coombs test	Positive	Negative
ESR (mm/hour)	13	0-20
Anti-Scl-70	Negative	Negative
Anti-Jo-1	Negative	Negative <0.9
Anti-centromere B	Negative	Negative <0.9

**Figure 1:**
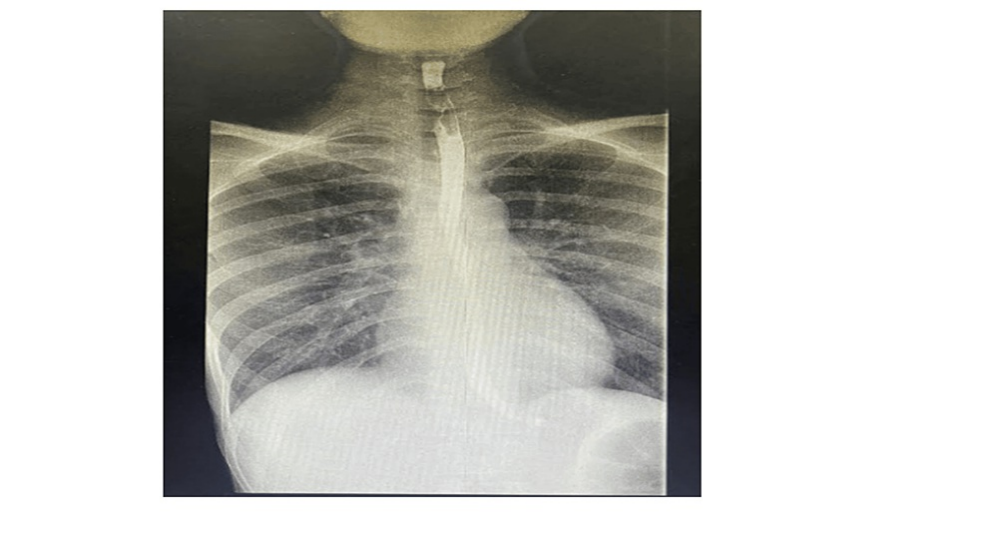
A case report with a barium swallow.

**Figure 2:**
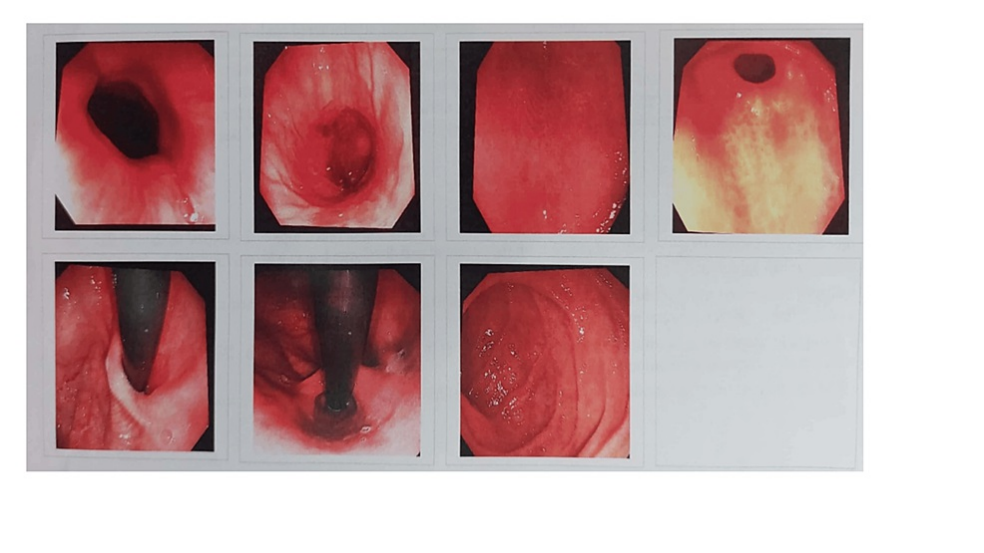
A case report with colonoscopy.

During admission, the patient underwent a barium swallow test, which revealed delayed gastric emptying (Figure 1). Her upper and lower scopes were normal (Figure 2), and her biopsies were negative. An eating disorder was excluded via a complete psychiatric evaluation. Malignancy was also excluded by negative pan-CT scans. Infection was ruled out due to unremarkable septic screens and cultures.

The patient’s positive Coombs test and delayed gastric emptying in barium swallow suggested an autoimmune disease, which seemed likely due to the patient’s age and rheumatological history.

The patient’s serology workup was positive for antinuclear antibody (ANA) and anti-roll and showed low C3 and C4 levels. She was negative for rheumatoid factor (RF), anti-Smith, and anti-cyclic citrullinated peptide (anti-CCP). The patient was thus diagnosed with lupus. According to the European League Against Rheumatism (EULAR) classification criteria, lupus is indicated by the presence of antibody markers, particularly when these markers appear in two organ systems. Our patient presented with three clinical criteria for lupus: joint involvement, oral ulcers, and non-scarring alopecia. Additionally, there were indications of leukopenia and autoimmune hemolysis. She also presented with two immunological criteria, resulting in a total score of 21 (Table 1).

Treatment was started immediately; the patient was administered 25 mg of steroids twice daily, 200 mg of hydroxychloroquine daily, and 5,000 IU of vitamin D with calcium weekly. She was also given proton pump inhibitors to alleviate her symptoms; manometry studies were conducted on an outpatient basis.

The patient’s clinical condition and eating improved, leading to weight gain and improved general health. She was discharged and instructed to follow up with the rheumatology and swallowing department. At a two-week follow-up, the patient’s dysphagia and odynophagia had improved; she had also gained 3 kg.

## Discussion

SLE has a wide range of uncommon presentations reported in the literature due to its multi-organ involvement of unknown etiology [1], the European League Against Rheumatism (EULAR)/American College of Rheumatology (ACR) diagnostic criteria for SLE do not include gastrointestinal signs, apart from oral ulcers, which may be observed during the clinical examination [1].

A prospective study in the United States conducted from 2018 to 2020 used high-resolution manometry (HRM) to examine 1,003 patients with this rheumatological condition. Of these, 9% had gastrointestinal involvement, 2.7% had systemic sclerosis, 2% had rheumatoid arthritis, and 1% had SLE. Furthermore, 61% had esophageal motility disorders. Three categories of these disorders were most prevalent: 28% of patients had absent contractility, 21% had inefficient esophageal motility, and 12% had esophagogastric outflow blockage. This study also found that individuals with esophageal problems had larger upper esophageal sphincters, lower distal contractile integrals, poorer bolus clearance, and more hiatal hernias than healthy controls [2].

In a multicenter cohort study conducted in 2018, 389 patients who presented with early manifestations of SLE were compared to 227 patients who presented with SLE mimickers. The study's results showed a significant correlation (*P* > 0.001) between dysphagia and early SLE manifestation; however, the study did not state dysphagia as the main presenting symptom [3].

The gastrointestinal tract is involved in 40% of SLE patients. Most of these cases are linked to vasculitis activity, autoimmune tissue damage, complications of organ dysfunction, thromboembolic symptoms of antiphospholipid antibody syndrome, infections, decreased saliva production, or adverse drug reactions due to long-term use [4,5]. Moreover, 20% to 70% of dysphagia-related complaints are linked to heartburn, chest pain, odynophagia, or regurgitation with a motility disorder [5].

The history of esophageal abnormalities has been extensively researched in previous literature [6]. Sultan et al. were the first to report that the number of motility diseases was increasing [7]. Castrucci et al., Lapadula et al., and a Mexican study found hypokinetic processes mostly affecting the upper third of the esophagus, with a normal lower esophageal sphincter, in three distinct types of manometry studies [8,9,10]. The pathophysiological mechanism for SLE-related esophageal dysmotility has been suggested to be either related to Auerbach plexus nerves or smooth muscle vasculitis [11].

Previous case studies report different manifestations of motility disorders in patients with SLE. These include esophageal epidermolysis bullosa acquisita, vagal nerve dysfunction caused by a focal CNS lesion in the vagus nerve nuclei (leading to the need for a temporary gastric feeding tube), and a link between dysphagia and chest pain brought on by esophageal spasms [11]. To improve assessment and ensure appropriate treatment, patients first undergo a barium swallow evaluation before they are subjected to further manometry. When a motility disorder is suspected, the patient might undergo an endoscopy with an additional tissue biopsy to check for vasculitis, Helicobacter pylori infection, or ulceration [11].

Unfortunately, due to a dearth of evidence-based data on responses to treatment in SLE patients and since immunosuppressant medications may also initiate symptoms by predisposing patients to opportunistic infections, motility disorders in SLE patients are still not well understood. However, other diagnostic studies may help identify a specific etiology, which could support management. PPI or metoclopramide along with lifestyle and diet modifications can be effective initial therapies, as many SLE patients experience gastroesophageal reflux disease (GERD) [4,12]. Nitrates and calcium channel blockers can be used to treat esophageal spasms, and steroids may be used to treat SLE vasculitis. However, no research has yet aimed to determine the best immunosuppressant agent for patients with SLE [12].

## Conclusions

Esophageal involvement is known to occur in some SLE patients over the course of the disease. Despite its rarity, SLE should be included in the differential diagnosis for any patient who exhibits the first predominant clinical indication of esophageal problems. Future research should explore the various types of dysphagia in more detail and measure the effectiveness of available treatments.

## Figures and Tables

**Figure 3:**
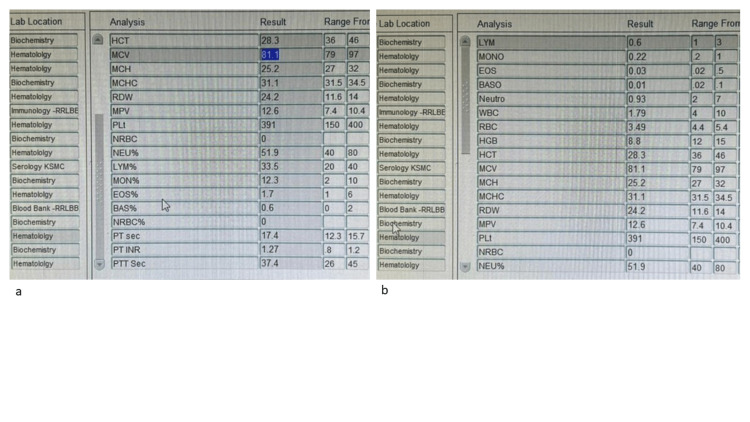
(a) and (b) Test reports of hematology laboratory tests.

**Figure 4:**
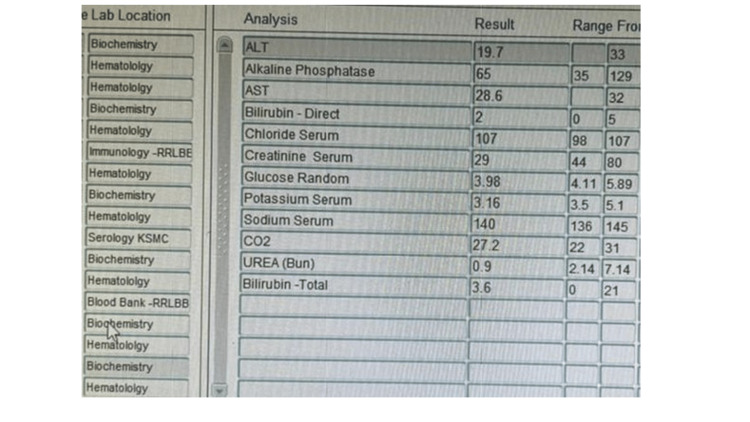
Test report of biochemistry laboratory findings.
